# Species-Specific Differential AhR Expression Protects Human Neural Progenitor Cells against Developmental Neurotoxicity of PAHs

**DOI:** 10.1289/ehp.0901545

**Published:** 2010-06-22

**Authors:** Kathrin Gassmann, Josef Abel, Hanno Bothe, Thomas Haarmann-Stemmann, Hans F. Merk, Kim N. Quasthoff, Thomas Dino Rockel, Timm Schreiber, Ellen Fritsche

**Affiliations:** 1 Department of Molecular Toxicology, Institut für umweltmedizinische Forschung gGmbH, Heinrich Heine University, Düsseldorf, Germany; 2 Department of Dermatology, University Hospital, RWTH Aachen, Aachen, Germany

**Keywords:** AhR, AhR knockout, DNT, neural progenitor cells, neurospheres, PAH

## Abstract

**Background:**

Because of their lipophilicity, persistent organic pollutants (POPs) cross the human placenta, possibly affecting central nervous system development. Most POPs are known aryl hydrocarbon receptor (AhR) ligands and activators of AhR signaling. Therefore, AhR activation has been suggested to cause developmental neurotoxicity (DNT).

**Objective:**

We studied the effects of AhR ligands on basic processes of brain development in two comparative *in vitro* systems to determine whether AhR-activation is the underlying mechanism for reported DNT of POPs in humans.

**Methods:**

We employed neurosphere cultures based on human neural progenitor cells (hNPCs) and wild-type and AhR-deficient mouse NPCs (mNPCs) and studied the effects of different AhR agonists [3-methylcholanthrene (3-MC), benzo(*a*)pyrene [B(a)P], and 2,3,7,8-tetrachlorodibenzo-*p*-dioxin (TCDD)] and an antagonist [3′-methoxy-4′-nitroflavone (MNF)] on neurosphere development. Moreover, we analyzed expression of AhR and genes involved in AhR signaling.

**Results:**

In contrast to wild-type mNPCs, hNPCs and AhR-deficient mNPCs were insensitive to AhR agonism or antagonism. Although AhR modulation attenuated wild-type mNPC proliferation and migration, hNPCs and AhR-deficient mNPCs remained unaffected. Results also suggest that species-specific differences resulted from nonfunctional AhR signaling in hNPCs.

**Conclusion:**

Our findings suggest that in contrast to wild-type mNPCs, hNPCs were protected against polycyclic aromatic hydrocarbon–induced DNT because of an absence of AhR. This difference may contribute to species-specific differences in sensitivity to POPs.

Persistent organic pollutants (POPs) bioaccumulate through the food chain and may cause adverse effects on human health and the environment. Main substance classes are polycyclic aromatic hydrocarbons (PAHs), such as 3-methylcholanthrene (3-MC) and benzo(*a*)pyrene [B(a)P], dioxins such as 2,3,7,8-tetrachlorodibenzo-*p*-dioxin (TCDD), and polychlorinated biphenyls (PCBs). Because of their lipophilicity, POPs cross the human placenta, exposing the fetus to the contaminant body burden of the mother. This may result in adverse health outcomes, including effects on central nervous system (CNS) development (reviewed by Wormley et al. 2004). One potential source for *in utero* exposure to POPs, especially PAHs, is maternal cigarette smoking during pregnancy (Polanska et al. 2009; Rodgman et al. 2000). An inverse relationship between maternal smoking during pregnancy, offspring intelligence (intelligence quotient), and cognitive ability has been described in numerous cohort studies (Batty et al. 2006; Fried et al. 1998; Olds et al. 1994). Additionally, behavioral problems and psychiatric disorders in offspring have been associated with smoking during pregnancy (Fergusson et al. 1998; Thapar et al. 2003; Weissman et al. 1999). In addition to smoking, *in utero* exposure to PCBs and/or dioxins has been associated with cognitive deficits in children (reviewed by Grandjean and Landrigan 2006; Jacobson and Jacobson 1997; Patandin et al. 1999). It has been proposed that etiological mechanisms involve the POP-activated aryl hydrocarbon receptor (AhR), an evolutionary highly conserved member of the basic helix-loop-helix/Per-ARNT-Sim (bHLH/PAS) family of transcription factors (reviewed by Kakeyama and Tohyama 2003). Ligand binding to this cytosolic receptor induces nuclear translocation (Gasiewicz and Bauman 1987) and heterodimerization with another bHLH/PAS protein, ARNT (AhR nuclear translocator) (Reyes et al. 1992). The resulting complex recognizes and binds specific DNA sequences (i.e., dioxin-responsive elements) within gene promoter regions and modulates subsequent transcription of AhR-dependent genes (Fujisawa-Sehara et al. 1987). Many POPs, including PAHs and dioxins, are known AhR ligands and activators of AhR signaling. It has therefore been proposed that AhR activation causes developmental neurotoxicity (DNT) (Williamson et al. 2005). This hypothesis is supported by studies in invertebrate (*Caenorhabditis elegans*) and vertebrate (zebrafish, chicken, rat, monkey) species in which dioxins and related compounds cause morphological abnormalities of the brain or deficits in cognition and/or behavior (Henshel et al. 1997; Hill et al. 2003; reviewed by Kakeyama and Tohyama 2003; Qin and Powell-Coffman 2004). Whether AhR activation is the underlying mechanism for the reported DNT effects after POP exposure in humans is not known.

To investigate potential species-specific differences, we employed comparative *in vitro* test systems for brain development based on neurosphere cultures from human and mouse neural progenitor cells (hNPCs and mNPCs, respectively). These three-dimensional cell systems mirror basic processes of fetal brain development such as proliferation, migration, differentiation, and apoptosis. Moreover, they detect developmental neurotoxicants *in vitro* (Fritsche et al. 2005; Moors et al. 2007, 2009). We report here that, in contrast to wild-type mNPCs, hNPCs and AhR-deficient mNPCs were insensitive to AhR agonism or antagonism due to nonfunctional AhR signaling, which suggests that humans are protected from AhR-dependent, POP-induced DNT. Knowledge about such species-specific differences is of utmost importance regarding chemical testing and hazard assessment for humans.

## Materials and Methods

### Chemicals

The AhR antagonist 3′-methoxy-4′-nitroflavone (MNF) was kindly provided by G. Vielhaber (Symrise, Holzminden, Germany). Methylmercury chloride (MeHgCl) was obtained from Riedel-de Haën (Seelze, Germany), and TCDD was purchased from LGC Standards (Wesel, Germany). All additional chemicals used (unless otherwise noted) were purchased from Sigma-Aldrich (Munich, Germany) and were of the highest purity available.

### Cell culture

hNPCs used in this study were purchased from Lonza Verviers SPRL (Verviers, Belgium). Data presented in this study are based on experiments conducted on hNPCs obtained from a single male at gestational week 16. Similar results were obtained based on hNPCs from a second male at gestational week 18. For mouse neurosphere cultures, brains of wild-type and AhR-knockout (KO) C57/BL6 mice (Charles River Laboratories International, Wilmington, MA, USA) were removed at embryonic day (E) 15.5 (E15.5) to E17.5 and transferred to phosphate-buffered saline. Age of the embryos was determined according to the staging criteria of Theiler, in which E16 correspond to Theiler stage 24 (Bard et al. 1998). Brains of embryos were dissected, transferred to Dulbecco’s modified Eagle’s medium (DMEM) and mechanically dissociated. Trypsin/EDTA solution was added, and the suspension was incubated for 30 min at 37°C in a humidified atmosphere. Afterward, the tissue suspension was triturated to obtain a single-cell suspension, which was centrifuged at 800 rpm for 5 min. Pellets were resuspended and plated in 10-cm petri dishes. AhR deficiency of mice (Fritsche et al. 2007) was confirmed by polymerase chain reaction (PCR). Data for wild-type mNPC were derived from four independent preparations, and data for AhR-deficient mNPCs were obtained from two different preparations. The animals were treated humanely and with regard for alleviation of suffering.

Both hNPCs and mNPCs were cultured in proliferation medium [DMEM and Hams F12 (3:1) supplemented with B27 (Invitrogen GmBH, Karlsruhe, Germany), 20 ng/mL epidermal growth factor (EGF; Biosource, Karlsruhe, Germany), 20 ng/mL recombinant human fibroblast growth factor (FGF; R&D Systems, Wiesbaden-Nordenstadt, Germany), 100 U/mL penicillin, and 100 μg/mL streptomycin] in a humidified 92.5% air/7.5% CO_2_ incubator at 37°C in suspension culture. Differentiation was initiated by growth factor withdrawal in differentiation medium [DMEM and Hams F12 (3:1) supplemented with N2 (Invitrogen), 100 U/mL penicillin, and 100 μg/mL streptomycin] and plating onto poly-d-lysine (PDL)/laminin–coated chamber slides.

### Cell viability

We measured cell viability using a lactate dehydrogenase (LDH) assay (CytoTox-One; Promega, Madison, WI, USA) according to the manufacturer’s instructions. Briefly, supernatants of treated cells from the migration, mRNA expression, and proliferation assessments were collected at the respective time points and incubated 2:1 with the CytoTox-One reagent for 4 hr prior to detection of fluorescence (excitation, 540 nm; emission, 590 nm). Complete lysis of cells with the included lysis buffer for 2 hr at room temperature served as a positive control.

### Proliferation assays, cell cycle analyses, and migration assay

Proliferation was assessed with a combination of CellTiter-Blue Assay (Promega), which measures mitochondrial reductase activity, and microscopic determination of sphere diameter as described previously (by Moors et al. 2009). We analyzed cell cycle by fluorescence-activated cell sorting (FACS). Therefore, neurospheres were exposed to the different chemicals for 48 hr, dissociated to single cells with Accutase, fixed with paraformaldehyde, and stained with propidium iodine (Moors et al. 2009). Spheres cultured in proliferation medium with 20 ng/mL EGF and FGF served as positive controls, and spheres cultured without these growth factors served as negative controls. Migration distance was measured microscopically after 48 hr as previously described by Moors et al. (2007, 2009). Spheres exposed to 1 μM MeHgCl for 48 hr served as positive controls.

### Quantitative real-time PCR

NPCs were treated under differentiating conditions with 10 μM 3-MC or 1 nM TCDD, or with 0.1% dimethyl sulfoxide (DMSO) as the solvent control. After indicated time points, RNA was prepared with the Absolutely RNA Microprep Kit (Stratagene, La Jolla, CA, USA) according to the manufacturer’s instructions. Real-time reverse-transcriptase PCR (RT-PCR) was performed using LightCycler instrumentation (Roche, Mannheim, Germany) with QuantiTect SYBR green PCR Master Mix (Qiagen, Hilden, Germany) as previously described (Fritsche et al. 2007). Conditions for PCR amplifications were as follows: initial 15 min at 95°C; 40 cycles of 15 sec at 94°C for denaturation, 25 sec of primer annealing, 30 sec at 72°C for elongation; and 2 sec at 72°C for fluorescence detection. Intron-spanning primers were designed using PRIMER 3 Software (Rozen and Skaletsky 2000) to reduce genomic amplification. Primer sequences for human *ACTB* (*hACTB*), *hAhR*, *hAhR* repressor (*hAhRR*), human cytochrome P450 1A1 (*hCYP1A1*), and *hCYP1B1* were described by Fritsche et al. (2005). Other primer sequences are as follows: *hARNT*, forward (F), CCCTAGTCTCACCAATCGTGGAT; reverse (R), GTAGCTGTTGCTCTGATCTCCCAG; *hC-MYC*, F, ACCACCAGCAGCGACTCTGA; R, TCCAGCAGAAGGTGATCCAGACT (56°C annealing); mouse *Actb* (*msActb*), F, CTACAATGAGCTGCGTGTGG; R, TAGCTCTTCTCCAGGGAGGA (60°C annealing); *msAhR*, F, GACAGTTTTCCGGCTTCTTG; R, CGCTTCTGTAAATGCTCTCGT (60°C annealing); *msArnt,* F, TGCCTCATCTGGTACTGCTG; R, GAACATGCTGCTCACTGGAA (58°C annealing); *msAhRR*, F, GTTGGATCCTGTAGGGAGCA; R, AGACCAGAGGCTCACGCTTA (60°C annealing); *msCyp1a1*, F, GGCCACTTTGACCCTTACAA; R, CAGGTAACGGAGGACAGGAA (54°C annealing); *msCyp1b1*, F, ACATGAGTTTCAGTTATGGCC; R, TTCCATTCACTGCTGAGAGC (58°C annealing); *msc-myc*, F, TGTCCATTCAAGCAGACG; R, GCATTTTAATTCCAGCGCATAG (54°C annealing). Expression levels were normalized to the expression of β-actin. We evaluated gene expression using the cycle threshold (Ct) value from each sample. Calculations are based on the ΔΔCt method (Livak and Schmittgen 2001). For determining absolute copy numbers, we used product-specific standards amplified from cDNA to generate standard curves. A target gene was considered to be quantifiable if the ratio of the copy number target gene to the copy number for β-actin × 1,000 exceeded 0.001.

### Western blotting

Proteins were isolated from hNPC or HepG2 human hepatocellular liver carcinoma cells as described by Moors et al. (2007). Whole-cell lysates (40 μg) were separated by 10% SDS-PAGE and transferred to an Amersham Hybond-P-membrane (GE Healthcare, Chalfont St. Giles, UK). The membrane was blocked in Tris-buffered saline containing 0.01% Triton (vol/vol) and 5% nonfat dry milk (wt/vol) for 1 hr at room temperature, followed by incubation in the same buffer containing a polyclonal anti-AhR antibody (1:2,000; Enzo Life Sciences, Loerrach, Germany) or an anti-GAPDH antibody (1:100,000; Biozol, Eching, Germany) overnight at 4°C. As secondary antibodies we used enhanced chemiluminescence (ECL) anti-rabbit IgG or ECL anti-mouse IgG horseradish peroxidase–linked antibodies (both 1:5,000; GE Healthcare). Chemiluminescence signals were detected with X-ray film.

### Statistics

All results are presented as mean ± SEM of at least two independent experiments. We used analysis of variance combined with Dunnett’s post hoc test for multifactor analyses (concentration effects), and Student’s *t*-test for two-group comparisons (treatment vs. control). Significance was set at *p* < 0.05 (Hays 1994).

## Results

### AhR agonism or antagonism does not cause cytotoxicity in hNPCs and mNPCs

Cells were exposed to the AhR agonists 3-MC (10 μM; a synthetic model compound for AhR-activating metabolized PAH), B(a)P (10 μM; a metabolized, environmentally relevant PAH), TCDD (1 nM; a nonmetabolized, environmentally relevant PAH), or the AhR antagonist MNF (1 μM; model compound). We treated hNPCs and mNPCs for 6, 12, 24, or 48 hr (differentiation conditions). hNPCs were also treated for up to 14 days and mNPCs were treated for up to 7 days under proliferation conditions. Exposure times for cytotoxicity assays thereby correspond to the duration of treatments in the proliferation, migration, and gene expression assays. We chose concentrations based on known AhR activation/antagonism in other cell types. None of the exposures induced cytotoxicity (LDH release) in hNPCs or mNPCs [see Supplemental Material, Figure 1 (doi:10.1289/ehp.0901545)].

### Proliferation of mNPCs, but not hNPCs, is inhibited by AhR blockage

**Figure 1:**
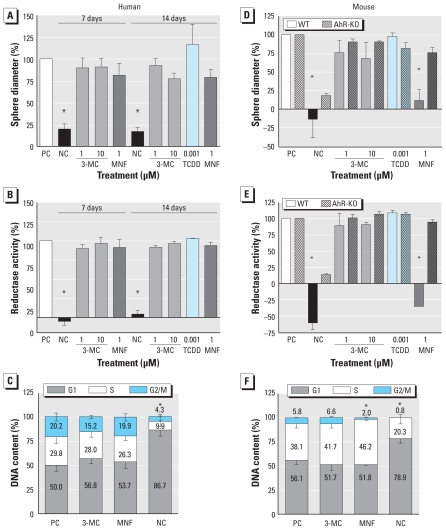
MNF inhibits proliferation of mNPCs (*D–F*) but not hNPCs (*A–C*). Abbreviations: NC, negative control (0.1% DMSO without added growth factors); PC, positive control (0.1% DMSO plus 20 ng/mL each of EGF and FGF); WT, wild type. After exposure of NPCs to 3-MC (1 μM and 10 μM), TCDD (0.001 μM), or MNF (1 μM) for 7 (mNPCs) or 14 (hNPCs) days, sphere diameters and mitochondrial reductase activities of human (*A, B*) and WT and AhR-KO mouse (*D, E*) neurospheres were measured; results are reported as percentages of corresponding measurements in PCs. The results for each end point were plotted in separate x,y-diagrams, and the gradient of the linear regression curve was assessed; data represent the mean gradient ± SEM of two (AhR-KO) to four independent experiments (5–6 spheres/exposure). Cell cycle phase distributions (values are shown within or above bars) of dissociated, fixed, and propidium iodine–stained hNPCs (*C*) and mNPCs (*F*) analyzed by FACS. Data represent the mean ± SEM of three independent experiments after 48-hr exposure to 10 μM 3-MC or 1 μM MNF. *p < 0.05 compared with PCs.

Figure 1A and B shows quantification of the proliferation assays (sphere diameter and CellTiter-Blue Assay, respectively) of human neurospheres after exposure to 1 and 10 μM 3-MC, 1 nM TCDD, or 1 μM MNF. We found no significant disparities in proliferation among cells with different exposures after 7 or 14 days. We verified this using hNPCs derived from a second individual (data not shown). The average diameter of the human neurospheres (excluding the negative controls cultured without growth factors) increased from 444 μm ± 15 μm to 653 μm ± 33 μm (at 7 days) and 829 μm ± 17 μm (at 14 days). The lack of exposure effects on proliferation was confirmed by FACS analyses for DNA content using propidium iodine staining (Figure 1C). We found a cell cycle distribution typical of proliferating cells in approximately 2.5% of all NPCs, as described previously (Moors et al. 2009). After 48 hr of exposure, most proliferating cells were in G0/G1-phase (53.5% ± 3.4%), whereas 28% ± 1.8% were in S-phase, and 18.4% ± 2.8% cells were in G2/M-phase, independent of exposure conditions.

We monitored proliferation of mNPCs over only 7 days because of a size-restricted halt in sphere growth beyond that time. In contrast to the hNPCs, we observed significant inhibition of mNPC proliferation after 7 days of exposure to 1 μM MNF. Although control spheres and 3-MC– and TCDD-exposed spheres grew from an average diameter of 386 μm ± 23 μm to 551 μm ± 85 μm, MNF-exposed spheres remained at 405 μm ± 78 μm after 7 days. This was due to AhR inhibition, because proliferation of AhR-deficient neurospheres was not affected by MNF treatment (Figure 1D,E). Effects of MNF on mNPC proliferation were confirmed by FACS analyses for DNA content using propidium iodine staining (Figure 1F). As for hNPCs, we found a cell cycle distribution typical of proliferating cells in approximately 2.5% of all cells. In the positive control (solvent plus growth factors), 56.1% ± 4.6% of these cells were in G0/G1-phase, 38.1% ± 4.3% were in S-phase, and 5.8% ± 0.4% cells were in G2/M-phase after 48 hr of exposure. 3-MC–treated cells showed a similar distribution. However, MNF treatment reduced the number of cells in G2/M-phase to 2.0% ± 0.4%, whereas the distribution of cells in G0/G1- and S-phase remained relatively constant at 51.8% ± 0.2% and 46.3% ± 0.4%, respectively.

### Neural cell migration is affected by AhR stimulation in mNPCs but not hNPCs

**Figure 2:**
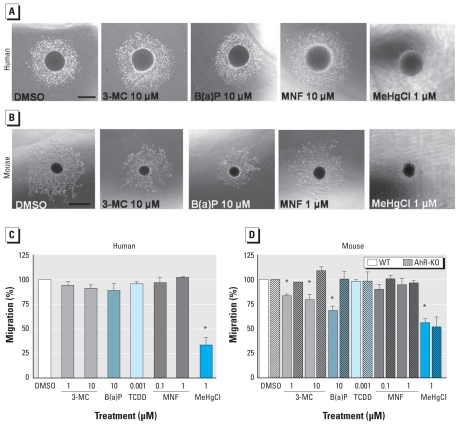
AhR agonists shorten wild-type mNPC but not hNPC migration. hNPCs (*A* and *C*) and wild-type (*B* and *D*) and AhR-KO (*D*) mNPCs were exposed to 3-MC, B(a)P, TCDD, MNF, or MeHgCl (PC) during differentiation for 48 hr. Migration distance from the edge of the sphere to the furthest outgrowth was measured. Data represent means ± SEs of two (AhR-KO) to five independent experiments (5–8 spheres/exposure). Bar = 500 μm. *p < 0.05 compared with 0.1% DMSO.

Next, we investigated whether 3-MC, B(a)P, TCDD, or MNF influences hNPC and mNPC migration. We exposed differentiating neurospheres to 1 and 10 μM 3-MC, 10 μM B(a)P, 1 nM TCDD, 0.1 μM MNF, or 1 μM MNF for 48 hr; 1 μM MeHgCl served as a positive control for inhibition of migration (Moors et al. 2009). Independent of exposures, all human neurospheres adhered to the PDL/laminin matrix and migrated radially out of the sphere with an average migration distance of 739 μm ± 61 μm after 48 hr (Figure 2A,C). This was verified using hNPCs from a second individual (data not shown).

In contrast to the results in hNPCs, AhR activation reduced migration distance in wild-type mNPCs by 16% ± 5% (1 μM 3-MC), 21% ± 13% (10 μM 3-MC), and 32% ± 10% [10 μM B(a)P] compared with solvent controls, whereas migration was comparable with controls after exposure to the AhR antagonist MNF (Figure 2B,D). This effect can be attributed to AhR activation because migration of AhR-deficient neurospheres was not disturbed by 3-MC or B(a)P (Figure 2D). Interestingly, TCDD did not disrupt wild-type mNPC migration despite AhR activation (Figure 2D). This might be due to the fact that, in contrast to 3-MC and B(a)P, TCDD is hardly metabolized and thus does not produce reactive intermediates.

### AhR-dependent gene transcription

**Figure 3:**
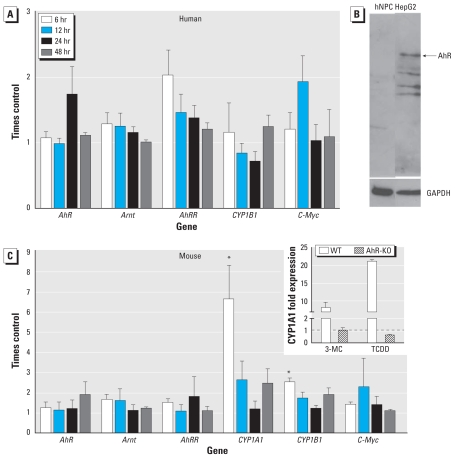
Induction of mRNA expression of the indicated genes in hNPCs (*A*) and mNPCs (*C*) analyzed 6, 12, 24, or 48 hr after treatment with 10 μM 3-MC under differentiating conditions. The inset in *C* shows *Cyp1a1* expression 6 hr after 1-nM TCDD or 10-μM 3-MC exposure in wild-type (WT) and AhR-KO mNPCs. Copy numbers of the respective genes were normalized to β-actin after and are expressed as relative induction compared with vehicle control (0.1% DMSO); data are mean ± SEM of two (AhR-KO) to four independent experiments; note the different *y*-axis scales in *A* and *C*. (*B*) hNPC lysates (40 μg protein/lane) analyzed for AhR expression by Western blot using an AhR-specific antibody and HepG2 cell lysates (10 μg protein/lane) as the positive control. A GAPDH-specific antibody was used as a loading control; a representative example is shown. *p < 0.05 compared with vehicle control.

AhR-dependent gene transcription is inducible only in mNPCs, and not in hNPCs because of low abundance of AhR and ARNT transcripts and absence of AhR protein in human cells. Because 3-MC, B(a)P, and MNF did not influence hNPC viability, proliferation, or migration but did modulate proliferation or migration of mNPCs, we determined *AhR* and *ARNT* mRNA expression after exposure to these compounds in hNPCs and mNPCs under proliferating and differentiating conditions. *AhR* and *ARNT* mRNAs were expressed at very low copy numbers in hNPCs (0.6–2.5 and 13/1,000 copies β-actin, respectively) and higher copy numbers in mNPCs (20–63 and 716–1,045/1,000 copies β-actin, respectively) (Table 1). In addition, *CYP1A1* expression was undetectable in untreated hNPCs. That β-actin was in this case valid to use as a housekeeping gene was demonstrated by normalization of proliferating versus differentiating NPCs to three additional housekeeping genes [*RPL27*, *RPL30*, *OAZ1*; see Supplemental Material, Figure 2 (doi:10.1289/ehp.0901545)]. Expression of the AhR target genes *AhRR*, *CYP1A1*, *CYP1B1*, and *c-myc* was not significantly induced by 10 μM 3-MC after 6, 12, 24, and 48 hr of differentiation in hNPCs (Figure 3A). We obtained comparable results for hNPCs from a second individual after 6 hr of treatment (data not shown). In contrast, 6 hr of exposure to 10 μM 3-MC significantly induced *Cyp1a1* and *Cyp1b1* mRNA in wild-type mNPCs to levels 6.6 ± 1.7 and 2.5 ± 0.25 times higher than controls, respectively (Figure 3C). Although 1 nM TCDD did not disturb neural migration, it induced AhR signaling in wild-type mNPCs, increasing *Cyp1a1* expression 21 times relative to controls (Figure 3C, inset).

Comparison of mRNA expression levels between hNPCs and mNPCs showed that genes belonging to the AhR machinery and AhR-dependent genes were generally expressed in higher copy numbers/1,000 copies β-actin in mNPCs than in hNPCs (Table 1). Lack of AhR protein in hNPCs was confirmed by Western blot (Figure 3B). These results demonstrate that AhR signaling pathway gene products mediate the effects of the AhR agonists 3-MC and B(a)P and the AhR antagonist MNF on proliferation and migration in mNPCs, because AhR-deficient mNPCs are protected against these effects (Figure 3C, inset).

## Discussion

The development of cell-based, nonanimal testing strategies for hazard assessment of chemicals is currently one of the most important tasks in toxicological research. In this regard, it is most important to choose appropriate model systems that are truly predictive for humans (Krewski et al. 2009; National Research Council 2007). Human tumor cell lines that are easily accessible in large quantities bear the restriction that they do not represent cellular metabolism and signal transduction of normal cells. In contrast, primary cells are often obtained as *ex vivo* cultures from rodents. Such primary cultures are regarded as superior to tumor-derived cells. However, species-specific differences limit their application. One example of how rodent primary cells can indeed misclassify hazards for humans is provided by this study, which shows mouse-derived primary cells to be more susceptible to AhR modulation than their human counterparts. With regard to chemical testing, it is critical to be aware of such differences in order to avoid over- or underestimating hazards that chemicals pose to humans, and thereby protect human health and allow industry production and development of chemicals at the same time.

Specifically, in this study we discovered that proliferation of hNPCs was not affected by AhR agonists (3-MC, TCDD) or the AhR antagonist MNF (Figure 1A–C), whereas proliferation of wild-type mNPCs was completely blocked by AhR antagonism (Figure 1D–F). Results of the proliferation analysis in hNPCs are in contrast to previous findings obtained in human liver or neuroblastoma tumor cells that showed exogenous AhR activation by TCDD or other AhR ligands to inhibit cell proliferation and induce cell cycle arrest (Jin et al. 2004; Marlowe and Puga 2005). In contrast, high AhR content has been reported to promote proliferation in a human MCF breast cancer cell line that was blocked by the AhR antagonist MNF or selective AhR knockdown via small interfering RNA (Wong et al. 2009). In human umbilical vascular endothelial cells, AhR activation by 3-MC also exerted antiproliferative effects, as in the tumor cell lines (Pang et al. 2008). These data demonstrate that effects of AhR activation on cell cycle progression versus cell cycle arrest vary among different cell types, as well as between tumor and nontumor cells.

In contrast to hNPCs, proliferation of mNPCs was completely blocked by MNF (Figure 1D–F). Reiners et al. (1999) reported that cell proliferation was also inhibited in the murine hepatoma cell line 1c1c7 by flavone and α-naphthoflavone treatment and in AhR-KO cells. Moreover, mouse embryonic fibroblasts from AhR-KO mice grew more slowly than did wild-type cells (Elizondo et al. 2000). In contrast, AhR-KO animals show accelerated proliferation in different organs, such as skin, hair follicles, and liver blood vessels. Thus, as in humans, AhR effects on proliferation in rodent cells are also cell type dependent, and as our data indicate, cell type specificity is not consistent among different species.

Besides progenitor cell proliferation, migration is another essential process in brain development. To address the role of the AhR in this process, we employed the neurosphere migration assay (Moors et al. 2007). AhR modulation by 3-MC, B(a)P, TCDD, or MNF did not affect hNPC migration (Figure 2A,C). These results were in contrast to the findings of two previous studies of human tumor cells that showed increased motility and migration of MCF-7 cells after TCDD and 3-MC treatment based on a scratch assay (Diry et al. 2006) and a transwell migration assay (Seifert et al. 2009). In contrast, we showed that AhR stimulation reduced migration of mNPCs (Figure 2B,D). This is supported by an earlier *in vivo* study in which prenatal exposure to the AhR ligand 7,12-dimethylbenz[*a*]anthracene disrupted cerebellar cytoarchitecture in rats (Kellen et al. 1976). Conversely, immortalized mouse mammary fibroblasts from AhR-null mice had decreased migration capacities in culture and inhibition of signaling pathways that regulate cell migration, including focal adhesion kinase and mitogen-activated protein kinase ERK1 (extracellular-signal regulated kinase-1) (Mulero-Navarro et al. 2005). That ERK-dependent pathways are also necessary for normal migration of hNPCs was recently shown by our group (Moors et al. 2007). However, AhR does not seem to determine ERK-dependent migration in hNPCs, because migration distance did not change in the presence of AhR modulators (Figure 2A,C) but was impaired by ERK inhibition (Moors et al. 2007). Taken together, existing data on AhR-dependent migration imply that, as for proliferation, modulation of cell migration differs among cell types and species.

To address the underlying reason for the observed species-specific differences in response to AhR modulation between hNPCs and mNPCs, we quantified copy numbers of genes that belong to the AhR machinery as well as genes that are AhR regulated. Both hNPCs and mNPCs express *AhR*, *ARNT*, *AhRR*, *CYP1B1*, and *c-Myc* (Figure 3A,C). However, *AhR* and *ARNT* copy numbers were close to detection limit in hNPCs (and 8–100 times lower than in mNPCs), and hAhR protein was not detectable by Western blot; consequently, only mNPCs expressed quantifiable amounts of *Cyp1a1* and responded to 3-MC treatment with a time-related induction of *Cyp1a1* and *Cyp1b1* mRNA (Figure 3). Moreover, low human AhR ligand affinity, which is approximately 10 times lower than C57/BL6 mouse AhR-ligand attraction (Harper et al. 1988), has been attributed to an amino acid substitution in the human AhR ligand-binding domain (Ema et al. 1994). This mutation is also responsible for divergent toxic potencies of TCDD between responsive C57/BL6 and nonresponsive DBA mouse strains (Ema et al. 1994). The irresponsiveness of hNPCs toward AhR modulation was hence likely due to very low *AhR* expression combined with a human low-affinity receptor for ligand binding. Because several polymorphisms have been identified in the human *AhR* gene, interindividual differences in ligand responsiveness between hNPCs from different donors cannot be excluded. However, even though single AhR polymorphisms (e.g., at codon 517 or 570) found in people of African descent were under suspicion to affect the expression of single genes such as *CYP1A1*, it is widely accepted that none of the AhR polymorphisms described so far is of any functional consequence regarding the overall outcome of AhR response (Connor and Aylward 2009; Harper et al. 2002).

Species differences similar to those reported here were observed in a comparative study of human and mouse palate organ cultures (Abbott et al. 1999). This earlier work was driven by the facts that TCDD induces cleft palate in mouse embryos and that the risk for humans to develop such malformations upon *in utero* exposure to TCDD or related compounds was not known. Abbott et al. (1999) concluded that it seems highly unlikely that human embryos are exposed to sufficient amounts of TCDD to cause interruption of palatal differentiation because human palate organ cultures expressed several hundred times less *AhR* mRNA than did the mouse cultures, and human palates required 200 times more TCDD to produce a cleft palate *in vitro* than did the respective mouse model. Our results support those findings in a different organ system, the developing brain, and suggest that studies on AhR-dependent DNT in C57/BL6 mice *in vivo* overestimate the risk of disturbances of human brain development resulting from AhR activation. Our data also imply that the use of toxic equivalency factors, which correspond to the relative potency of a chemical to generate AhR-mediated effects compared with TCDD, based on data derived from rodents for risk assessment of POPs may not necessarily be useful for humans.

How can the epidemiological evidence for POP-related DNT in humans be explained if the AhR is not involved in chemically induced DNT? Kodavanti (2005) comprehensively reviewed three alternative mechanisms for how POPs might interfere with human brain development. First, POP-induced changes in neurotransmitters such as dopamine or serotonin could affect learning, memory, and other functions. Because these end points extend beyond the basic processes of brain development, the basic “neurosphere assay” (Breier et al. 2009) cannot detect such changes. Second, PCBs were found to alter intracellular phosphokinase C (PKC) signaling and Ca^2+^ homeostasis in rodents. Because PKC modulation by the inhibitor BisI (bisindolylmaleimide I) or the stimulator PMA (phorbol 12-myristate 13-acetate) causes inhibition and stimulation of hNPC migration, respectively (Moors et al. 2007), it is unlikely that the POPs used in this study affected PKC signaling. Third, effects on thyroid hormone (TH) balance might contribute to POP-induced DNT. Some POPs can elicit direct biological effects on target organs such as the brain by interfering with cellular TH signaling *in vivo* and *in vitro* (Fritsche et al. 2005; Schreiber et al. 2010; Zoeller and Crofton 2000). Because the neurosphere assay is able to detect endocrine disruption of TH signaling by PCBs and polybrominated diphenyl ethers (Fritsche et al. 2005; Schreiber et al. 2010), this mechanism is hence unlikely to be involved in AhR-independent DNT of “classical” AhR ligands such as 3-MC or TCDD in humans. The most likely mechanism through which POPs interfere with human brain development is via systemic effects on TH homeostasis resulting from direct effects on the thyroid gland and decreased synthesis of TH, reduced blood TH levels secondary to enhanced TH metabolism, or displacement of natural ligand (thyroxine) binding to the TH plasma transport protein transthyretin. Evidence that TCDD also affects systemic TH balance in humans was reported by Baccarelli et al. (2008), who found that maternal exposure in the highly TCDD-exposed Seveso cohort was associated with effects on neonatal thyroid function (Baccarelli et al. 2008). However, we cannot exclude the possibility that AhR expression in fetal brains increases with gestational age, resulting in AhR-mediated toxicity of POPs in NPCs at later gestational stages.

In summary, we show that, in contrast to mNPCs, hNPCs are protected against PAH-induced DNT and that this difference may be explained by the absence of AhR in hNPCs. An accumulating body of evidence now indicates that human AhR signaling is less operative than AhR function in most laboratory animals. This knowledge should be taken into account for risk assessment of TCDD and related xenobiotics in humans.

## Figures and Tables

**Table 1:** Comparison of human and mouse mRNA copy numbers/1,000 copies of β-actin in proliferating and 24-hr differentiating NPCs.^a^

	Human		Mouse		Mouse:human ratio	
Gene	Prolif	Diff	Prolif	Diff	Prolif	Diff
*AhR*	2.45	0.62	20.08	63.21	8.19	102.12
*ARNT*	12.95	13.55	716.26	1045.07	55.31	77.13
*AhRR*	0.94	0.61	79.21	387.55	84.24	638.85
*CYP1A1*	< 0.001	< 0.001	18.63	31.64	ND	ND
*CYP1B1*	0.01	0.06	146.24	834.91	16407.23	14990.75
*C-MYC*	5559.59	3017.13	14835.55	15967.72	2.67	5.29

Abbreviations: Diff, differentiating; ND, not detectable; Prolif, proliferating.

a Data represent at least three independent experiments. The mouse:human ratio is shown to compare human and mouse mRNA expression levels.
